# Experimental demonstration of multi-dimensional resources integration for service provisioning in cloud radio over fiber network

**DOI:** 10.1038/srep30678

**Published:** 2016-07-28

**Authors:** Hui Yang, Jie Zhang, Yuefeng Ji, Yongqi He, Young Lee

**Affiliations:** 1 State Key Laboratory of Information Photonics and Optical Communication, Beijing University of Posts and Telecommunications, No.10 Xitucheng Road, Beijing, 100876, China; 2 State Key Laboratory of Advanced Optical Communication Systems and Networks, Peking University, Beijing, 518129, China; 3 Huawei USA, Plano, TX 75023, USA

## Abstract

Cloud radio access network (C-RAN) becomes a promising scenario to accommodate high-performance services with ubiquitous user coverage and real-time cloud computing in 5G area. However, the radio network, optical network and processing unit cloud have been decoupled from each other, so that their resources are controlled independently. Traditional architecture cannot implement the resource optimization and scheduling for the high-level service guarantee due to the communication obstacle among them with the growing number of mobile internet users. In this paper, we report a study on multi-dimensional resources integration (MDRI) for service provisioning in cloud radio over fiber network (C-RoFN). A resources integrated provisioning (RIP) scheme using an auxiliary graph is introduced based on the proposed architecture. The MDRI can enhance the responsiveness to dynamic end-to-end user demands and globally optimize radio frequency, optical network and processing resources effectively to maximize radio coverage. The feasibility of the proposed architecture is experimentally verified on OpenFlow-based enhanced SDN testbed. The performance of RIP scheme under heavy traffic load scenario is also quantitatively evaluated to demonstrate the efficiency of the proposal based on MDRI architecture in terms of resource utilization, path blocking probability, network cost and path provisioning latency, compared with other provisioning schemes.

With the rapid evolution of internet of things and mobile internet, the fifth generation of mobile networks (5G) has become a next major phase of mobile telecommunications^1^, which is able to implement a future with both user-services and machine-type communications where access to information and sharing of data is available anywhere and anytime^2^. It is well-known that a very high system throughput of up to 1000 times today’s throughput will be required by 2020 for large-scale 5G networks with the development of system scale and user requirement^3^. To cope with the problem, the cloud radio access network (C-RAN) is a paradigm introduced by operators^4^ which aggregates all base stations computational resources into a cloud baseband unit (BBU) pool, while the distributed radio frequency signals are collected by remote radio head (RRH) and transmitted to the cloud platform through optical transmission^5^. A RRH executes radio frequency functionalities of a base station, while the BBU handles base band processing functions. It aims to reduce capital and operating expenditure and enhance real-time cloud computing while offering better services^6^.

Recently, the interaction between RRH and BBU or resource schedule among BBUs in cloud have become more frequent and complex due to the exponentially growing number of mobile internet users, drastically increased mobile applications and more data-rich Internet content^7^. Due to the diversity and hugeness of the user demands, a large number of high-performance services have presented the high burstiness and high heterogeneity characteristics, especially for multi-rate traffic in fronthaul. The simplified optical transmission has become the bottleneck of information interaction in traditional C-RAN structure. The heavy-duty interaction caused by services promotes the networking demand among RRHs and BBUs and forces to form elastic optical fiber switching and networking^8^ due to the characteristics of high bandwidth, low cost and transparent transmission. On the other hand, evolving from wavelength division multiplex, elastic optical network (EON) with flexible gird is achieved by taking advantage of the orthogonal frequency division multiplexing (OFDM)^9^, which can allocate necessary spectrum resources with a fine tailored granularity for various user connection demands (e.g., sub-wavelength and super-channel traffic), and offer cost-effective and highly-available connectivity channels^10^,^11^,^12^. As the typical technology, the EON meets the interconnection requirement and thus forming the enhanced C-RAN connected with EON which is called cloud radio over fiber network^13^ (C-RoFN).

However, the radio network, optical network and BBU cloud have been decoupled from each other in such network, so that the resources of them have been controlled independently. The accommodation of service request can be setup respectively in the form of segmentation among those networks^14^. The traditional architecture cannot efficiently enough implement the resource optimization and scheduling for the high-level service guarantee due to the communication obstacle among them^15^. It cause that the concept of user-centered cannot be realized in 5G scenario. Additionally, the software defined networking (SDN) enabled by OpenFlow protocol can be as a unified control architecture^16^,^17^,^18^,^19^,^20^ to provide centralized control over different resources for the joint optimization of services with a global view, and provide maximum flexibility for operators^21^,^22^,^23^,^24^,^25^. Thus, it is very important to applying the SDN to implement the control and optimization of the resource assignment in such C-RoFN environment.

The cross stratum optimization using SDN between optical network and application stratum resources is proposed to meet the QoS requirement in our previous work^26^,^27^,^28^,^29^ within the scope of transport network. On the basis of it, in this paper, we aim at the new access network scenario (i.e., C-RoFN), and extend to report a study on a novel multi-dimensional resources integration (MDRI) for service provisioning in cloud radio over fiber network. The MDRI adapts the SDN orchestration to provide a unified control over multiple dimensional resources through the SDN controllers for the joint optimization of end-to-end services with a global view. The motivations for MDRI architecture in C-RoFN can break the limit among radio, optical and processing unit domains, implement the multiple layer integration and cross stratum optimization with SDN orchestration, which can allocate and optimize multiple dimensional resources efficiently in a control manner of open system. The MDRI architecture can provide the basis of resource scheduling and a unified topology consolidating cross multiple stratums. Additionally, based on the proposed architecture, a resources integrated provisioning (RIP) scheme using an auxiliary graph for MDRI in C-RoFN scenario is introduced to globally schedule the route considering multiple dimensional resources, e.g., radio frequency, spectrum, power, distance and computational unit. The MDRI can enhance the responsiveness to dynamic end-to-end user demands and globally optimize radio frequency, optical network and processing resources effectively to maximize radio coverage. The feasibility of the proposed architecture is experimentally verified on OpenFlow-based enhanced SDN (eSDN) testbed^30^. The performance of RIP scheme under heavy traffic load scenario is also quantitatively evaluated to demonstrate the efficiency of the proposal based on MDRI architecture in terms of resource utilization, path blocking probability, network cost and path provisioning latency, compared with other provisioning schemes. The results indicate this scheme has excellent performance and constitutes a promising candidate for future cloud radio over fiber network.

## MDRI architecture for cloud radio over fiber network

**Figure 1:**
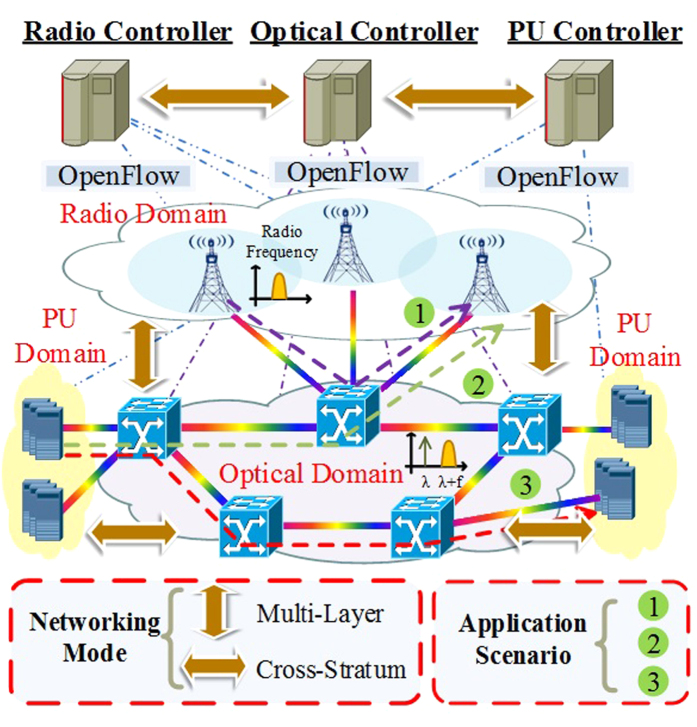
The architecture of MDRI for software defined C-RoFN. The logical relationship among the networking modes and application scenarios for C-RoFN is shown in Figure. The networking mode for multi-dimensional resources integration extends in two directions. One is from the perspective of resource form. Optical and processing resources are interconnected cross optical network and processing stratums along the east-west direction. It leads to the interconnection and networking of heterogeneous resources cross stratums in *latitudinal direction*, which is established as “*heterogeneous-cross-stratum*”. The other is the relationship of carrying capacity. The related entity with small granularity of switching can be abstracted as the high-layer network (e.g., radio network), while the related entity with large granularity of switching should be abstracted into the low-layer network (e.g., EON). The interconnection and networking of multiple layers are established along *longitudinal direction*, which is called “*multi-layer-carried*”. Based on the above-mentioned networking mode, three kinds of applications in this architecture are formed, i.e., the interaction between antennas (e.g., coordinated multiple points transmission (CoMP)), the service from antenna to PU and resource schedule among clouds (e.g., virtual migration inter-PU).

The multi-dimensional resources integration (MDRI) architecture uses the idea of SDN for reference in C-RoFN. The MDRI can implement the multiple layer integration and cross stratum optimization based on OpenFlow-enabled C-RoFN with SDN orchestration, which can allocate and optimize the resources of radio, optical network and processing interweaved with each other efficiently in a control manner of open system. The MDRI architecture for C-RoFN is illustrated in Fig. 1. The EON is used to interconnect the cloud processing units (PU), which deployed network and processing (e.g., virtual machine, computing and storage) stratum resources respectively. The distributed antennas are interconnected and converged into EON, which allocates the customized spectrum with finer granularity for radio signals. Note that, multiple dimensional resources in C-RoFN have been divided into three stratums, which includes radio resource, optical spectrum resource, and cloud processing resource stratums. Each resource stratum can be software defined with OpenFlow protocol (OFP) and controlled by a radio controller (RC), an optical controller (OC) and a PU controller (PC) respectively in a unified manner. To control heterogeneous networks for MDRI with OFP, OpenFlow-enabled antenna and bandwidth-variable optical switch with OFP agent software are required, which are referred to as OF-antenna and OF-BVOS respectively, as proposed in ref. 9. The motivations for MDRI architecture in software defined C-RoFN are twofold. Firstly, the MDRI can emphasize the cooperation between the RC and OC to overcome the interworking obstacles deriving from multi-layer overlaid networks and it effectively realizes vertical integration. Secondly, in order to provide the end-to-end QoS, multiple stratum resources can be merged through controllers’ interaction with horizontal merging, while achieving global cross stratum optimization of optical network and processing resources.

**Figure 2:**
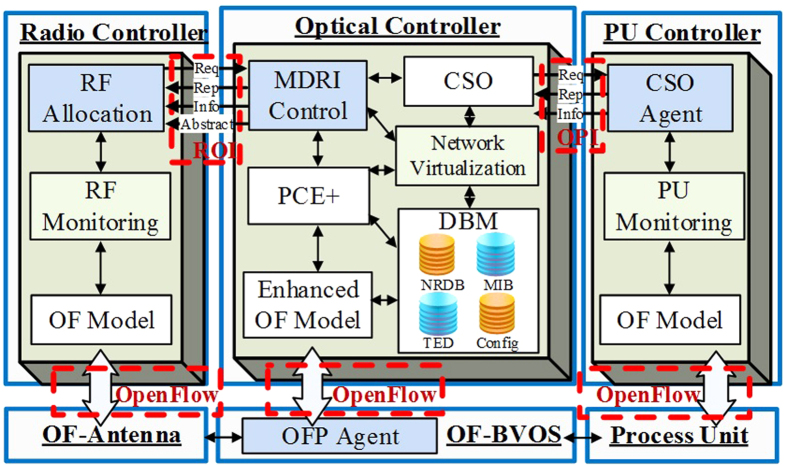
The functional models of radio, optical and PU controllers. The responsibilities and interactions among these functional building blocks of three controllers are shown in figure.

To obtain the functional architecture described above, the radio, optical and PU controllers have to be extended to support the MDRI and shown in Fig. 2. Note that, the OFP agent software embedded in OF-BVOS maintains optical flow table and models node information as software and maps the content to control the physical hardware^9^. In the OC, the network virtualization module is responsible for virtualizing the required optical network resources and interworks the information to perceive the EON through the enhanced OpenFlow module. Meanwhile, the radio frequency monitoring module in RC obtains and manages virtual radio resource in antenna, while the PC obtains PU resource information periodically or based on event-based trigger through a PU monitoring module. When the service request is arrival, the MDRI control module can perform the RIP scheme using auxiliary graph. The information can be interacted between the RC and OC through radio-optical interface (ROI). After completing the proposed scheme, the MDRI control module can decide which nodes and corresponding links are selected as the path for service accommodation. Then it provides this request to path computation element (PCE) module in turn, including the request parameters (e.g., latency and bandwidth), and eventually returning a success reply including the information of the provisioned path. Here, the PCE is capable of computing a network path or route based on a network graph, and of applying computational constraints. To conveniently perform the path computation with the CSO of optical and PU processing stratum resources, the OC can be interacted with PC through optical-PU interface (OPI). After receiving the processing resources information from the PC, the end-to-end path computation can be completed in PCE module considering CSO of the optical and PU resources. Note that, the various strategies can be alternative in PCE module as a plug-in. The enhanced OpenFlow module and RF allocation module perform continuous spectrum and radio frequency assignment for the computed path and provision the path by using OFP. When the path is setup successfully, the information of the path is conserved into the data base management (DBM) in the OC, which can interact with network virtualization module and store the virtual network and PU resources for MDRI. Once service request arrives, the CSO agent in PC provides the computing resource utilization periodically.

## Methods

### Problem statement

In the proposed multiple stratum architecture, the service should be provided by using multiple dimensional resources to accommodate with low latency and high bandwidth. Traditionally, the radio path calculation has been performed in radio domain, while optical controller is responsible for computing the spectral path and spectrum allocation. If those dimensional resources are maintained separately along one path, i.e., the path calculation has been divided into serval parts, it is hard to obtain the globally optimal result on the resource utilization or propagation delay. Extremely, the service cannot be reached with just considering the local optimization. From the perspective of user, the *radio path* (*RP*) computation and radio frequency assignment consider to reuse the radio frequencies as much as possible without causing interference between adjacent antennas. In the optical transport stratum, the *spectral path* (*SP*) is expected to use fewer spectrum resources to carry more radio signals under the optical stratum constraints, e.g., spectrum continuity and contiguity. Under heavy traffic load scenario, EON can offer highly-available, cost-effective and energy-effective connectivity by provisioning a sub-wavelength or super-wavelength level SP. For instance, if the user equipment is placed on the edge of cells, i.e., located at the same distance and channel environment to two adjacent antennas, so both of base stations can serve for the user. Which node can be chosen for signal provisioning to the device has become confused with considering one stratum. Therefore, we study a novel resources integrated provisioning (RIP) scheme that is essential for the proposed architecture to support the service accommodation using the mixed path with both radio and optical stratum resources. Such path uses both radio frequency and EON resources through radio and optical stratums, which is called *mixed path* (*MP*). It performs more effective service provisioning, i.e., utilizing less resources and enhancing user’s QoS and network performance.

**Figure 3:**
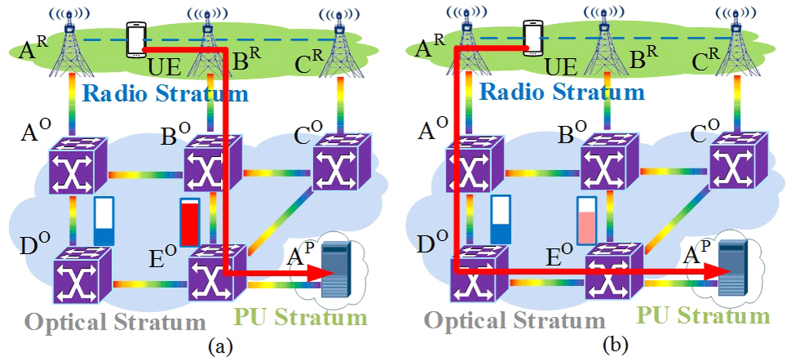
Schematic diagram of different schemes: (**a**) traditional provisioning scheme with considering multiple dimensional resources separately, (**b**) resources integrated provisioning scheme with multi-stratum unified.

**Figure 4:**
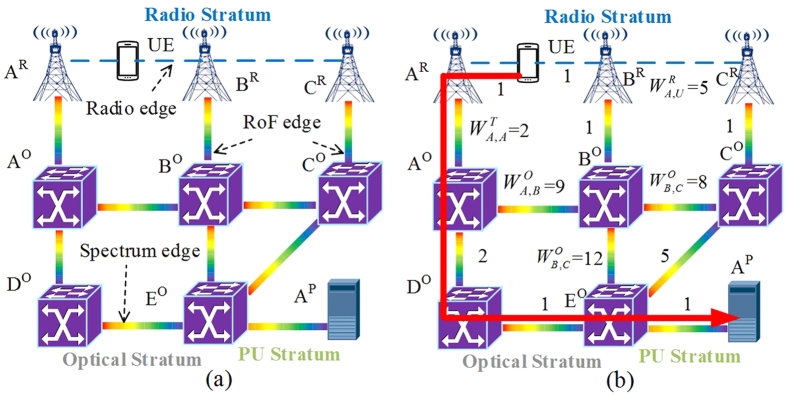
Illustration of auxiliary graph for RIP scheme: (**a**) auxiliary graph, (**b**) an example provisioning path using auxiliary graph.

Figure 3(a,b) show an example of RIP scheme in C-RoFN with a simple network topology, which contains three antennas, five optical switches and a PU. There is a user equipment in this instance between two antennas nodes *A*^*R*^ and *B*^*R*^, which can access either one of them for service. For simplicity, this example doesn’t consider the CoMP technology. A path from source node *UE* to destination PU *A*^*P*^ is used to accommodate the service, and radio signal is carried on fiber from antenna node *B*^*R*^. The path *UE*-*B*^*R*^-*B*^*O*^-*E*^*O*^-*A*^*P*^ is chosen due to the shortest route in the network. It means that the optical node *B*^*O*^ and *E*^*O*^ are the source and destination of related *SP* respectively, as shown in Fig. 3(a). It follows that the spectrum utilization of optical link *l*_*BE*_ have been very high and other requests from link *l*_*BE*_ will be blocked due to the resource exhaustion when the network is loaded heavily, and thus degrading the radio coverage and network performance. In such scenario, we also assume the switching is not currently supported in antenna. The proposed scheme doesn’t only consider the number of hops to selection the route, but also calculate the sum of edge weight. In fact, each edge of the auxiliary graph has its own weigh which can be calculated according to the Eqs 1, 2, 3, 4 (which are shown as follows). The principle of the path selection is to choose the one with sum of the minimum weight among the corresponding links, which is derived by Dijkstra’s algorithm considering the edge weight. The route of *UE*-*A*^*R*^-*A*^*O*^-*D*^*O*^-*E*^*O*^-*A*^*P*^ can be selected because it has the minimum sum of weight compared with other paths (we give the example in Fig. 4(b)). To solve such problem, the RIP scheme uses the *MP UE*-*A*^*R*^-*A*^*O*^-*D*^*O*^-*E*^*O*^-*A*^*P*^ with radio and spectrum resources to support the service, as shown in Fig. 3(b). The new antenna node *A*^*R*^ with the same distant as *B*^*R*^ can be found to access the *UE* for service fronthaul, while the corresponding optical link *l*_*AD*_ with lower spectrum resource usage in the optical stratum can provide the radio on the new *SP* effectively. Note that, the accessed antenna wouldn’t be changed once it serve for the user, while they are just the presumed examples in the instance. The new *MP* can enhance the network resource utilization with lower blocking effectively in the proposed scheme.

### Network modeling

We represent the software defined C-RoFN with MDRI as a weighted graph *G* (***V**, **V***^***′***^*, **L**, **L***′*, **F**, **F***′*, **A***), where ***V ***= {*v*_1_, *v*_2_, …, *v*_*n*_} and 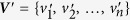 denote the set of OpenFlow-enabled optical switching and antenna nodes, respectively. In addition, ***L*** = {*l*_1_, *l*_*2*_, …, *l*_*n*_} and 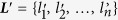 indicate the set of bi-directional fiber links between nodes in ***V*** and ***V***′. Also, ***F*** = {*s*_1_, *s*_2_, …, *s*_*F*_} and ***F***′ = 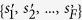 are the set of optical spectrum and radio frequency on each fiber link and ***A*** denotes the set of PU nodes, while *N*, *N*′, *L*, *L*′, *F*, *F*′ and *A* represent the number of optical and antenna nodes, links, the spectrum and radio slot and PU nodes respectively. For each service request from source *s* to destination *d*, it can be translated into the needed network and processing resources. Note that these resources contain the required network bandwidth *b* and processing resources *ar* in the analysis of network model for simplicity. We denote the *i*th service request described above as *SR*_*i*_ (*s*,*d*,*b,ar)*, while *SR*_*i+1*_ will arrive after service demand *SR*_*i*_ in the time order. According to the request and status of resources, the suitable *MP* can be provided as the path provisioning based on the scheme. In addition, some requisite notations and their definitions used in the study are listed in Table 1.

### Resources integrated provisioning scheme

In this study, we propose an auxiliary graph to implement RIP scheme according to its edge weights. An auxiliary graph, illustrated in Fig. 4(a), is constructed each time a new service request arrives. The nodes in each stratum of the auxiliary graph correspond to the nodes in the physical topology. The auxiliary graph is composed of the radio and optical stratums and three kinds of undirected edges, i.e., radio edges, RoF edges and spectrum edges.

There is a radio edge between the *UE* and antenna node in radio stratum if the signal can provide the link between them in the physical network. To measure the accommodation capacity of antenna, we consider the radio frequency utilization and the ratio of distance and power on the radio link for assessing the workload of antenna. Thus, for the service request, the radio edge weight *W*^*R*^_*i,j*_ between *UE* node *i* and *j* in recent time *t*_*0*_ is useful for assessing the antenna average recent occupation, which is expressed as the Eq. 1. Here, *RF*_*i,j*_ and *RF*_*0*_indicate the occupied radio frequency slots and total resources on the link, while *D*_*i,j*_ and *P*_*i,j*_ mean the distance and power between node *i* and *j* respectively. Since the dimensions of distance and power are various, we use parameter *q* to unify them with the same standard. Also, the adjustable parameter *k* is used to normalize the radio edge weight.


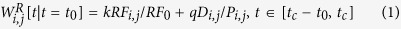


A RoF edge between antenna node at the radio stratum and its corresponding node in optical stratum represents the conversion from radio frequency to modulated optical spectrum. Note that, we use the simple amplitude modulation with double sideband to modulate the radio signal to optical spectrum for simplicity. So, its edge weight *W*^*T*^_*i,j*_ evaluates the cost of modulation, which is expressed as the Eq. 2. Note that, the radio parameters contain the symbol rate *B*_*i,j*_ and radio frequency *F*_*i,j*_ of current radio signal, and *a* indicates the normalized parameter.


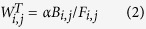


The optical stratum can be used to represent the spectrum resources, which is possibly used by new lightpaths that can carry the radio signal. Different from WDM network, to accommodate the new request successfully in EON, there must be at least *b* + *B* consecutive available sub-carriers in each fiber through the new *SP*, including the new requested bandwidth *b* as well as a guard band *B*. Also, with the consideration of spectrum continuity constraint, the spectrum of the new *SP* must be continuous in all the fibers through the path. From optical link’s perspective, the number of possible spectrum allocation status using the *s*th sub-carrier for all service bandwidth demands is described as *m*_*s*_ on link *l*_*i,j*_, while the connecting bandwidth of each possible provided status is represented as *b*_*k*_. So, the average bandwidth *b*^*s*^_*i,j*_ of all probable allocation status using the *s*th sub-carrier on *l*_*i,j*_ uses the equation below, in which the value of *b*^*s*^_*i,j*_ indicates the consecutiveness degree of the *s*th sub-carrier to the adjacent available spectrum.


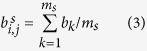


In addition, the number of sub-carrier occupation state change for neighboring sub-carriers on *l*_*i,j*_, defined as *v*_*i*_,_*j*_, is useful to estimate the degree of the spectrum fragmentation on one link. Particularly, the higher degree of fragmentation means it is more difficult to search consecutive spectrum on the link. In order to assess the spectrum utilization, the spectrum edge weight 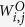 considers consecutive and fragmented degree of spectrum as shown in what follows. Here, the adjustable parameter *μ* normalizes the spectrum edge weight.


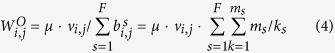


The RIP scheme for MDRI in C-RoFN that employs the auxiliary graph is described in Table 2. When new service demand arrives, the request *SR*_*i*_ (*s*,*d*,*b*,*ar*) arrives at the network, a corresponding auxiliary graph is established according to current network state in recent time *t*_*0*_. Note that the edge weights are calculated following the above equations to reflect the C-RoFN resources utilization. Based on the auxiliary graph, Dijkstra’s algorithm considering the edge weight is computed from source to destination nodes in multiple stratums network to select the path candidate. The principle of the path selection is to choose the one with sum of the minimum weight among the corresponding links. In fact, the topology for the RIP scheme contains multiple stratums network, i.e., radio, optical and PU stratums. The topology information in corresponding stratum has been collected and updated in related controller periodically. In the OC, the network virtualization module interworks the node and link information to perceive the EON, and consolidate the information to update the optical network topology. In addition, the radio frequency monitoring module in RC obtains and manages virtual radio resource in antenna, while the PC obtains PU resource information periodically or based on event-based trigger through a PU monitoring module. Through the ROI and OPI, the MDRI control module receives the abstracted radio and PU information which are provided from RC and PC respectively, and then calculates and seams the auxiliary graph topology for the RIP scheme. Note that, we use the private protocols using PCE communication protocol (PCEP) for reference which named radio-optical and optical-PU interfaces based on UDP message to extend the topology information delivery and route for RIP scheme. If the chosen path is *MP* (i.e., go through radio and optical paths), it determines which optical node should be the edge optical node to modulate the radio signal, and whether and how we should setup new lightpaths. The new lightpath can be established according to the selected spectrum edges including corresponding weight, and go through the fiber links with spectrum continuity constraint. The routing scheme with CoMP can be also performed in the auxiliary graph, which will be analyzed in another work due to space limitation. An example auxiliary graph for a new service request from node *UE* to node *A*^*P*^ is illustrated in Fig. 4(b). The weights of radio, RoF and spectrum edges are marked in the figure. When the request from node *UE* occurs, the example service *MP* using auxiliary graph is derived by Dijkstra’s algorithm from *UE* to node *A*^*P*^ with the minimum sum of corresponding edge weight (i.e., 1 + 2 + 2 + 1 + 1 = 7). This *MP* specifies that the new request can be carried on radio from *UE* to *A*^*R*^ in radio stratum, and then accommodated using a new *SP* from node *A*^*O*^ to *E*^*O*^ which is established with the spectrum resources in EON. Note that the *MP* uses a RoF edge *A*^*R*^*-A*^*O*^, i.e., an additional modulation when setting up the new lightpath. With the development of the network scale, the memory space and Dijkstra’s calculation time on the graph will increase and thus influencing the operational performance. Due to the development of network scale, the ultra-large network is divided into multiple domains for the operation and maintenance, since the control signaling storm will inevitably stress the performance of a single controller. The MDRI architecture with multiple controllers can deal with the information of multi-domain network. In such scenario, the auxiliary graph can be also divided into multiple domains maintained in corresponding controller respectively. In fact, the computation on the graph can be parallel performed when the network is large-scale. Intra-domain path calculation with single domain auxiliary graph is performed in one controller, while the inter-domain algorithm (e.g., BRPC) should be addressed to compute sparse path. Therefore, the proposed scheme has the scalability for multi-domain large-scale network.

## Results

**Figure 5:**
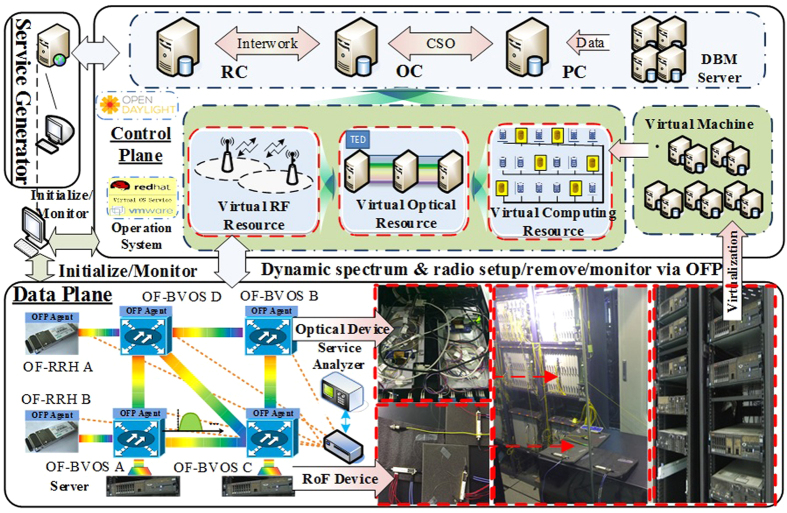
Experimental testbed for MDRI and demonstrator setup.

To evaluate the feasibility of the proposed architecture, we set up an EON with software defined C-RoFN based on our testbed, as shown in Fig. 5. In data plane, two analog RoF intensity modulators and detect modules are utilized, which driven by a microwave source working at 40 GHz frequency to generate double sideband. Four OpenFlow-enabled elastic ROADM nodes are equipped with Finisar BV-WSSs in the EON. We use Open vSwitch (OVS) as software OFP agent according to the API to control the hardware and interact between controller and radio and optical nodes. In addition, OFP agents are used to emulate other nodes in data plane to support the MDRI with OFP. The PU cloud and OFP agents are realized on an array of virtual machines created by VMware ESXi V5.1 running on IBM X3650 servers. The virtual operation system technology makes it easy to set up experiment topology for large scale extension. For OpenFlow-based MDRI control plane, the OC server is assigned to support the proposed architecture and deployed by means of three virtual machines for MDRI control, network virtualization and PCE strategy as plug in, while the RC server is used as radio frequency resource monitor and assignment. The PC server is deployed as CSO agent to monitor the computing resources from PUs. Each controller server controls the corresponding resources, while the database servers are responsible for maintaining traffic engineering database (TED), connection status and the configuration of the database. We deploy the service information generator related with the RC, which implements batch C-RoFN services for experiments.

**Figure 6:**
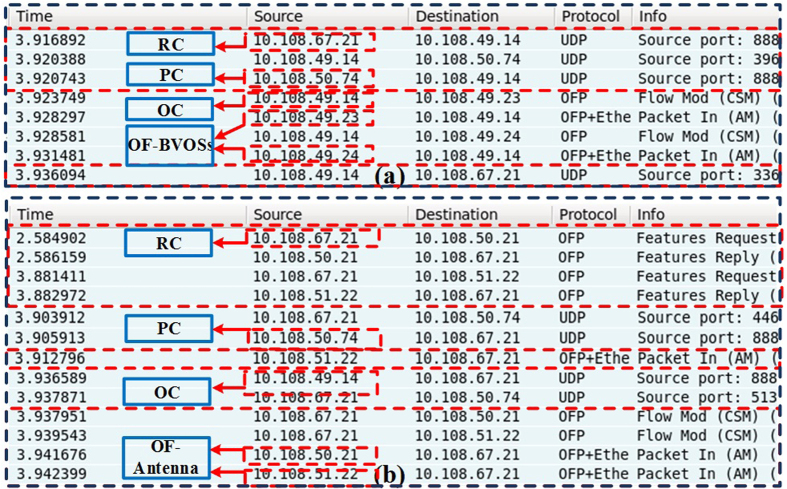
Wireshark capture of the message sequence for MDRI in (**a**) OC and (**b**) RC.

**Figure 7:**
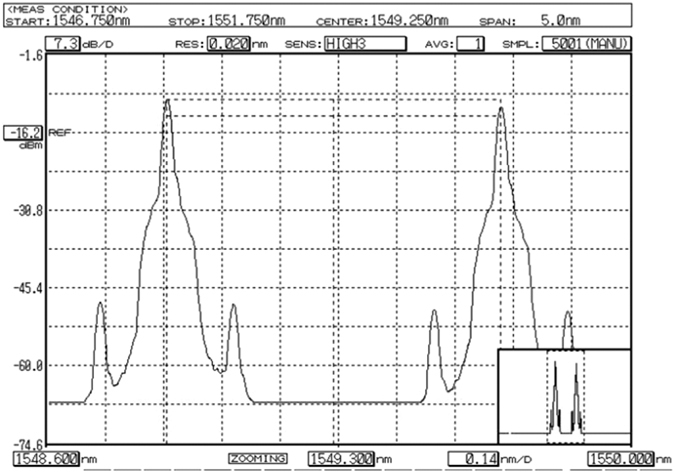
Filter output of spectrum.

Based on the testbed, we have designed and verified experimentally MDRI for service in C-RoFN. The experimental results are shown in Figs 6 and 7. Figure 6(a,b) present the signaling procedure for MDRI using OFP through a Wireshark capture deployed in OC and RC respectively. Note that existing OpenFlow messages have the original function, which are reused to simplify the implementation in this paper. The new messages types for C-RoFN will be studied and defined to support new functionalities in our future work. As shown in Fig. 6(a,b), 10.108.67.21, 10.108.50.74 and 10.108.49.14 denote the IP addresses of the RC, PC and OC respectively, while 10.108.49.23 and 10.108.49.24 represent the IP addresses of related OF-BVOSs respectively. The features request message is responsible for monitoring by regularly querying OF-BVOSs about the current status. The OC obtains the information from OF-BVOSs via features reply. When the service request arrives, the RC sends the request for MDRI via UDP message, where we use UDP message to simplify the procedure and reduce the performance pressure of controllers. After receiving the resources information from the interworking, the OC performs the RIP scheme to compute the paths considering multiple dimensional optimization of radio, elastic optical network and PU resources with auxiliary graph, and then reserve the optimal radio frequency, spectrum and processing resources for the service provisioning. After completing RIP, the OC and RC provision *SP* and assign the radio frequency to control the corresponding nodes via flow mod message. Receiving the setup success reply via packet in, the RC responds the MDRI success reply to PC and updates the computational usage to keep the synchronization. The spectrum of lightpath for analog C-RoFN is reflected on the filter profile, as shown in Fig. 7. The radio signals can be modulated on the spectral channel with MDRI.

## Discussion

We also adopt the backbone topology of continental US to evaluate the performance and scalability of MDRI based on RIP scheme under heavy traffic load scenario and demonstrate the efficiency of the proposal comparing it with the traditional radio-based scheme (RBS), optical-based scheme (OBS) and global evaluation scheme (GES)^31^ through virtual machines. The topology uses the backbone topology of continental US including 100 nodes and 171 links. It is illustrated as a hypothetical core fiber transport network indicative of the larger inter-exchange carriers of the continental United States^32^. The traditional RBS scheme carries the services to consider the radio frequency resource allocation in radio stratum, while the optical network and PU resource can be selected randomly to accommodate the service. The OBS scheme chooses the shortest lightpath from the random source antenna to destination node with the spectrum assignment in optical stratum. The GES can globally analyze the optical network and PU resource status cross stratums, and select the optimal PU node as the destination node according to the joint resource utilization. The service requests are setup with bandwidth randomly distributed from 500 MHz to 40 GHz, where the spectrum slots in EON is 6.25 GHz typically. The service processing usage in PU is selected randomly from 1% to 0.1% for each demand. The services present some statistical characteristic for some time when they arrive at C-RoFN. Based on the rules in statistics, if the demands have the fixed average instantaneous rate and arrive at C-RoFN randomly and independently, they can follow the Poisson process. In such scenario, each service demand’s duration and inter-arrival interval duration follow the negative exponential distribution. Also, several individual services may come to network uncertainly. They can be treated by the way of individual service classification. The individual service classification mixing with statistics analysis will be researched in our future work. In the experiment setting, we assume the services arrive at the network following a Poisson process and results have been extracted through the generation of 1 × 10^5^ demands per execution. The *α* and *μ* are adjustable weight parameters among radio, RoF and spectrum edges, which can impact the results of routing via auxiliary graph. For simplicity, we set the values of *k*, *q* and *t*_*0*_ as 0.5, 0.5 and 50 ms in the simulation settings. To analyze the proposed scheme in depth, we consider RIP scheme with different adjustable weight parameters in auxiliary graph. When values of *α* and *μ* is set as 0.6 and 0.4, the abbreviation of RIP scheme is referred to as RIP-0.6. We also use RIP-0.5 by parity of reasoning. In the emulation, we use these two typical values, i.e., RIP-0.5 and 0.6 to compare the performance for simplify. In this work, we evaluate the performance of MDRI with RIP scheme using the backbone topology of continental US including 100 nodes and 171 links, in terms of resource utilization, path blocking probability, network cost and path provisioning latency. In the practical network scenario, the network with 100 nodes is a large-scale topology which deploys the control plane. With the development of network scale, the ultra-large network should be divided into multiple domains for the operation and maintenance, since the control signaling storm will inevitably stress the performance of a single controller. If the scale of network exceeds 100 nodes, the MDRI architecture with multiple controllers (e.g., multiple OCs or RCs) can deal with the information of multi-domain network. The inter-domain algorithm (e.g., BRPC) should take the place of single domain algorithm (e.g., Dijkstra’s) to enhance the path calculation efficiency.

**Figure 8:**
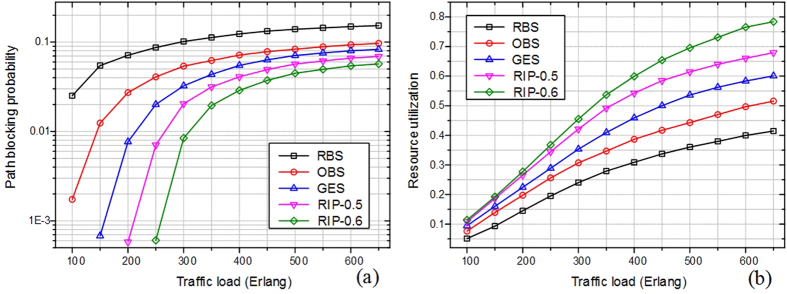
(**a**) Path blocking probability and (**b**) resource utilization among various schemes under heavy traffic load scenario.

**Figure 9:**
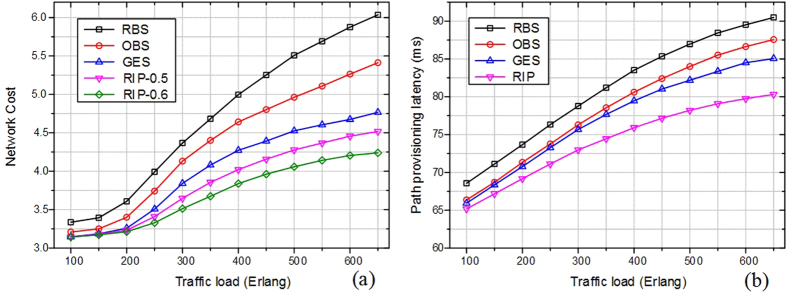
(**a**) Network cost and (**b**) path provisioning latency among various schemes under heavy traffic load scenario.

Figure 8(a) compares the path blocking probability among traditional RBS, OBS and GES and RIP schemes in backbone topology of continental US. It can be seen clearly that RIP scheme achieves better path blocking probability values as compared to the other schemes, especially when the network is heavily loaded. The reason is that, the RIP scheme avoids much traffic to be transferred into the heavy loaded antenna, where lots of services may be blocked or lost due to the queue overflow. In addition, consecutive and fragmented degree of spectrum are also considered in the proposed scheme using auxiliary graph. The selected path is more likely to be setup successfully during the spectrum allocation phase. Another phenomenon occurs that path blocking probability of RIP scheme reduces when the ratio values of radio and spectrum edges rise, i.e., RIP-0.5 and 0.6. The conversion from radio frequency to optical network is relatively scarce compared to sufficient spectrum resource. The higher ratio value means more RoF edge weights can be considered in auxiliary graph. The phenomenon is due to the fact that the service with higher ratio value can provide with more available radio and spectrum resources than that in lower ratio scenario. The comparisons on resource utilization among those schemes are shown in Fig. 8(b). Resource utilization reflects the percentage of occupied resources to the entire radio, EON and PU resources. As shown in figure, the RIP scheme can enhance the resource utilization remarkably compared to the other schemes. This is justified by the fact that RIP scheme can optimize the multiple dimensional resource at a global view and as such can yield higher resource efficiency. Figure 9(a) shows that the RIP scheme outperforms other schemes in the network cost, which is calculated according to the proportion of 2:1 using optical switch and antenna along the mixed path. The reason is much of elastic optical switches can be saved by using auxiliary graph. The performances among those schemes in terms of path provisioning latency are compared in Fig. 9(b). In the simulations, the latency reflects the average provisioning delay including antenna forwarding and optical transmission time. The RIP scheme significantly reduces the path provisioning latency compared to other schemes. That is because RIP scheme can reduce the congestion in the queue of services by optimal multi-dimensional resource usage. It allows saving a large amount of queuing delay. The other schemes use resources with considering one kinds of stratums with one dimensionality, and thus lead to longer queueing delays. This phenomenon is more obvious under heavy traffic because more requests need to be queued and the times of RBS, OBS and GES schemes increase, which will augment the delay time.

To meet the QoS requirement of 5G services, this paper reports a MDRI architecture for service provisioning in software-defined C-RoFN. Additionally, the RIP scheme is introduced for MDRI in the proposed architecture, which considers the auxiliary graph to calculate mixed path for service provisioning. The functional architecture and control modules are described in this paper. The feasibility of MDRI is verified on our OpenFlow-based eSDN testbed built by control and data planes. We also quantitatively evaluate the performance of RIP scheme under heavy traffic load scenario to demonstrate the efficiency of the proposal in terms of path blocking probability, resource utilization, and provisioning latency, and compare it with other relevant state of the schemes. The results indicate that the MDRI with RIP scheme can utilize multi-dimensional resources of radio frequency, optical network and processing effectively and enhance end-to-end user responsiveness of services, while leading to a reduced blocking probability coupled with cost savings. We hope the work presented in this paper will be beneficial for the industrial deployment of C-RAN in the near future. Our future MDRI work includes two aspects. One is to improve RIP scheme performance considering the time-varying parameters and consider the scalability issues of C-RoFN with multi-domain topology. The other is to implement the network virtualization and individual service classification mixing with statistics analysis for MDRI in C-RoFN on our OpenFlow-based testbed.

## Additional Information

**How to cite this article**: Yang, H. *et al*. Experimental demonstration of multi-dimensional resources integration for service provisioning in cloud radio over fiber network. *Sci. Rep.* **6**, 30678; doi: 10.1038/srep30678 (2016).

## Figures and Tables

**Table 1:** Notations and Definitions.

Symbol	Meaning
*s*	The source of service request
*d*	The destination of service request
*b*	The requested bandwidth of service request
*B*	The number of sub-carriers consumed by guard band
*l*_*i,j*_	The optical link between node *i* and *j*
*MP*_*i, j*_	The mixed path from node *i* to *j*
*SP*_*i, j*_	The spectral path from node *i* to *j*
*RP*_*i, j*_	The radio path between node *i* and *j*

**Table 2:** Resources integrated provisioning scheme.

Algorithm 1: Resources integrated provisioning scheme
**Input**: *G* (***V**, **V***′*, **L**, **L***′*, **F**, **F***′*, **A***), *SR*_*i*_ (*s*,*d*,*b*,*ar*)
**Output**: Provisioning *MP*
1: Construct auxiliary graph and calculate weights 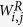 , 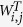 , 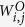 based on Eqs 1, 2, 3, 4 to corresponding edges at last *t*_*0*_ time.
2: Run Dijkstra’s algorithm from node *s* to *d* with the minimum sum of corresponding edge weight.
3: **if** no path is found **then**
4: Block the service request.
5: **else**
6: Route *R*_*i*_ according to the path found:
7: **if** the path includes radio edges **then**
8: Route the request in radio stratum network according to edge weight 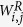 .
9: **end if**
10: **if** the path includes RoF and spectrum edges **then**
11: Setup the new *SP*s using the corresponding route and spectrum assignment based on selected spectrum and RoF edge weights 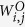 , 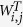 .
12: **end if**
13: Update the C-RoFN state and edge weights 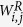 , 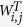 , 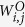 .
14: **end if**
15: Tear down all paths when request terminates.
16: Update the C-RoFN state and edge weights 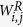 , 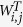 , 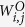 .
